# 1144. Evaluation of Procalcitnonin Usage in Neonates Presenting with Fever or Suspected Sepsis

**DOI:** 10.1093/ofid/ofab466.1337

**Published:** 2021-12-04

**Authors:** Hita Bhagat, Beech Burns, James Lewis, Diana Yu

**Affiliations:** 1 Oregon Health & Science University, Portland, Oregon; 2 Oregon Health and Science University, Portland, Oregon; 3 Oregon Health and Science University/Doernbecher Children’s Hospital, Portland, OR

## Abstract

**Background:**

Clinical evaluation alone is not effective in identifying serious bacterial infections (SBI) in neonates presenting with suspected sepsis and fever. A clinical decision making tool to aide in evaluating neonates presenting to the pediatric emergency department (PED) uses urinalysis, absolute neutrophil count (ANC), and procalcitonin (PCT) and together has high negative predictive value (NPV) for SBI. Use may decrease invasive testing, antibiotic exposure, and rates of admission. The tool was incorporated into hospital guidelines in October 2020. The purpose is to assess implementation and prediction of SBIs.

**Methods:**

This is a single-center quality improvement study at an academic medical center. Neonates less than 60 days presenting with fever or suspected sepsis were included in the baseline group from October 2019- March 2020 or the post-implementation group from October 2020- March 2021. Exclusion criteria were receiving antibiotics 48 hours before PED visit, pre-existing medical conditions, indwelling devices, soft-tissue infections, and ≤ 36 weeks gestation. Implementation and guideline compliance was assessed in neonates aged 29-60 days as the primary outcome. Secondary endpoints include initiation of empiric antibiotics, rates of admission, rates of re-presentation within 30 days, and rates of lumbar punctures in all included patients.

**Results:**

The baseline group had 29 patients and the post-implementation group had 35 patients who met inclusion/exclusion criteria. Baseline characteristics were similar with higher SBI rates in the post-implementation group having 8 SBIs while the baseline group had 4. There were 16 patients aged 29-60 days in the baseline (55%) and 17 in the post-implementation groups (49%). Complete labs were available for 9 patients (53%) and guideline compliance was 89%. NPV in neonates aged 0-60 days with negative urinalysis, ANC, and PCT was 100%. Rates of secondary endpoints were slightly higher in the post-implementation group along with higher rates of infections.

**Conclusion:**

High NPV in this small cohort is an indication for continued use of this tool in neonates presenting to the PED with suspected sepsis or fever. Further education to increase use and expansion to all neonates should be considered based on overall NPV and previous studies.

**Disclosures:**

**All Authors**: No reported disclosures

